# Fabrication of Dimples by Jet-ECM of Zr-Based Bulk Metallic Glasses with NaCl-Ethylene Glycol Electrolyte

**DOI:** 10.3390/mi14122196

**Published:** 2023-11-30

**Authors:** Cheng Guo, Jingwen He, Weizhen Zhuang, Kangsen Li, Duo Li

**Affiliations:** 1 College of Mechatronics and Control Engineering, Shenzhen University, Shenzhen 518060, China; hejingwen2021@email.szu.edu.cn (J.H.); 2310295112@email.szu.edu.cn (W.Z.); 2 State Key Laboratory of Ultra-Precision Machining Technology, Department of Industrial and Systems Engineering, The Hongkong Polytechnic University, Hung Hom, Hong Kong, China; kangsli@polyu.edu.hk; 3 Center for Precision Engineering, Harbin Institute of Technology, Harbin 150001, China; liduo@hit.edu.cn

**Keywords:** jet electrochemical machining (jet-ECM), Zr-based bulk metallic glasses (BMGs), NaCl-Ethylene Glycol electrolyte, surface integrity

## Abstract

Zr-based bulk metallic glasses (BMGs) possess unique mechanical and biochemical properties, which have been widely noticed. Jet electrochemical machining (jet-ECM), characterized by a high-speed jet, is a non-contact subtractive method with a high resolution and a high material removal rate (MRR). Past work on the electropolishing of Zr-based BMGs has indicated the feasibility of the NaCl-Ethylene Glycol (EG) electrolyte. In this research, the jet-ECM of Zr-based BMGs in the NaCl-EG electrolyte was studied to explore the dissolving mechanisms and surface integrity according to the voltage, pulse-on time and effective voltage time. The diameter, depth and surface morphologies of dimples were evaluated. The results showed that using this alcohol-based electrolyte led to a desirable surface morphology. The diameter and depth of the dimples varied with the voltage and the effective voltage time in a significantly positive proportional manner. Additionally, cases based on multiple parameter sets exhibited different stray corrosion severity. Afterward, machining performance can be enhanced in the next stage by tuning machining parameters to obtain microscale dimples with better quality.

## 1. Introduction

Zr-based bulk metallic glasses (BGMs) are an amorphous alloy fabricated under an ultra-cold solidification condition, and they possess a high glass-forming ability. Due to their distinctive microstructure, Zr-based BMGs exhibit superior characteristics compared to traditional materials, including excellent magnetic properties, corrosion resistance, wear resistance, hardness and toughness [1]. Moreover, the adjustment and control of the surface properties of BMGs to enhance their practical applications have been a subject of ongoing attention [2]. Currently, Zr-based BMGs can meet requirements for many high-performance components, such as fuel cell electrodes [3], sports facilities [4], medical devices [5,6,7] and micro transmission components [8,9]. To achieve high-precision dimensions and superior surface quality, the common approach for processing BMGs involves methods such as cutting, electrical discharge machining (EDM) and laser processing. In the course of the cutting process, the elevated hardness and toughness exhibited by Zr-based BMGs may result in tool wear, and achieving a mirror-like finish on the machined surface can be difficult [10]. 

EDM and laser processing, originating from thermal-physical mechanisms, result in a recrystallized layer on the surface of the processed BMGs due to melting and subsequent solidification [11,12,13]. This type of machining method would destroy the original amorphous state and worsen the usability of the Zr-based BMGs. 

Electrochemical machining (ECM) is a precision subtractive manufacturing technique based on the effective and controllable electrochemical dissolution of the anode. This special mechanisms of ECM bring multiple advantages [14], including a high machining rate irrespective of the workpiece hardness, high surface integrity and the avoidance of tool wear, heat-affected zones and residual stresses. In contrast to the aforementioned subtractive manufacturing methods, electrochemical machining can overcome these problems [15] because electrochemical dissolution is immune to the physical properties of Zr-based BMGs and maintains its original amorphous state. Jet electrochemical machining (jet-ECM) is a significant ECM variation. Differently, there is no cathode tool for the production of a negative imprint on the workpiece in jet-ECM [16]. During the process, the electrolyte is ejected from the cathode nozzle in a positive flow manner, and an electrical potential is applied between the workpiece and the nozzle. The high-flow-rate electrolyte can remove the scum produced by processing, and there is no concomitant effect on the processing surface [17]. Many researchers have already used this technology to create various microstructures, such as dimples and channels, which have been applied in aerospace, medical devices, biomedical applications and microelectronics. Liu et al. [18] investigated the machining performance of high-volume fraction SiCp/Al using an electrochemical jet with an NaCl electrolyte. They found that under the sustained impact of high-speed jets, the entrance diameter of the machined hole was approximately 3–4 times the size of the jet. Lu et al. [19] introduced a plasma in the electrochemical jet system to apply a sufficiently high voltage to tackle the issue of oxide layer formation in the electrochemical machining of chemically inert or passivating materials. This research augmented the range of materials that could be processed using jet electrochemical machining. Cheng et al. employed jet-ECM to remove the surface defects of the selective laser melting (SLM) formed parts, and the results were compared with the traditional sandblasting and sandpaper grinding surface treatment methods. After jet-ECM, the SLM surface defects were almost completely removed, forming a uniform microporous structure that interlocked with the nickel coating [20].

Due to the diverse electrochemical properties of the constituents in Zr-based BMGs, the selection of a suitable process for jet-ECM is highly complicated. Current research predominantly employs water-based salt electrolytes and alcohol-based salt electrolytes. However, water-based salt electrolytes lead to the formation of densely adhered passive films on the surface, hindering the electrochemical dissolution of Zr-based BMGs in water-based electrolytes. Many research findings have indicated that water-based salt electrolytes are unsuitable for achieving the uniform electrochemical dissolution of Zr-based BMGs [21,22,23]. In recent years, many researchers have begun to utilize NaCl-EG electrolytes for the electrochemical machining of Ti and its alloys [24,25]. Liu et al. introduced a kind of NaCl electrolyte into the ECM process of titanium alloy to enhance its machining performance. Comparative experiments on groove machining indicate that NaCl-EG electrolyte has better shaping accuracy compared to NaCl water-based electrolyte. In addition, experiments indicate that elevating the electrolyte temperature to some degree contributes to the miniaturization of geometric shapes in jet-ECM using NaCl-EG electrolyte [12]. Therefore, alcohol-based electrolytes can be employed for the electrochemical machining of Zr-based BMGs. Moreover, in alcohol-based electrolyte, Clˉ can actively electrochemically dissolve most metals due to its minuscule ionic radius, assimilating the different dissolution mechanisms between passivating and active components present in water-based electrolytes [26].

In this research work, jet-ECM was utilized to investigate the electroerosion of Zr-based BMGs in an NaCl-EG electrolyte. The study primarily delves into the impact of varying voltage, dwell time and pulse-on time on the dissolution mechanisms and surface integrity of Zr-based BMGs, in terms of diameter, depth and surface morphology. The experimental results demonstrated that voltage and effective voltage time have a significant impact on the sample morphology, especially on the diameter and depth of the dimples. In order to obtain better machining efficiency and machining quality, the reasonable selection of jet-ECM parameters is increasingly important. Moreover, alcohol-based solutions containing NaCl have proven to be highly effective in manufacturing multi-component alloys like Zr-based BMGs.

## 2. Materials and Methods

**Figure 1:**
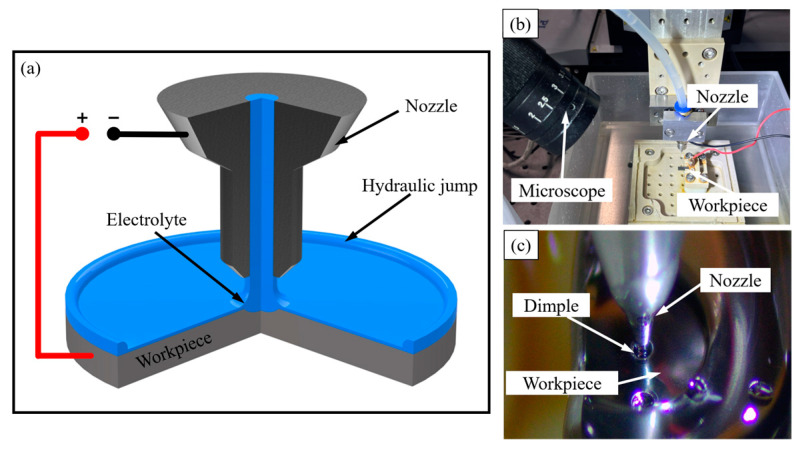
Experimental device schematic and physical drawings: (**a**) illustration of the jet-EJM principle; (**b**) physical device; (**c**) workpiece process.

Figure 1a illustrates the schematics of the jet-ECM system. The basic principle is that the electrolyte ejects from the nozzle with the inner diameter of 200 μm. The flow rate is lower in high-viscosity electrolytes, making the formation of hydraulic jump more challenging. The selection of an appropriate electrolyte viscosity should be based on the safety pressure (2.0 MPa) of various components. A HPLC pump is used to push the electrolyte with the flow rate of 52 mL/min. The viscosity effect hinders the electrolyte flow rate from reaching the set value, and measurements indicate that the actual flow rate of the electrolyte is 52 mL/min, so the electrolyte speed of the electrolyte can be calculated as 27.5 m/s. Under the impact of a high-flow electrolyte, the exposed surface area is 3.2 mm^2^. The electrolyte impacts the workpiece surface and a circular hydraulic jump form, within which a thin film of the electrolyte spreads on the surface. Jet-ECM confines the electrolyte column beneath the nozzle, where the current density is far greater than other regions with power on.

The experimental setup in Figure 1c depicts the nozzle configuration. The nozzle was amounted on a three-axis motorized stage from PI. The moving precision of the stage along each axis was 0.1 μm. The workpiece was fixed in an electrolyte tank with a bottom export. The gap between the workpiece and the nozzle was set to 200 μm to maintain enough current densities. The electrolyte was 1 mol/L NaCl-ethylene glycol solution. The workpiece material was a kind of amorphous alloy, Vit1 (Zr_41.2_Ti_13.8_Cu_12.5_Ni_10.0_Be_22.5_). The workpiece surfaces to be processed were ground by waterproof abrasive paper up to #5000 and cleaned ultrasonically with deionized water and ethanol.

The power supply system for experiments was a combination of a high-voltage amplifier and a signal generator. The current and voltage signals were samples by isolation probes. The effects of the dwell time and the applied voltage were investigated by altering the power supply. The direct current and pulse current (5 kHz) were used in the experiments to clarify the effects of waveforms. In order to evaluate the effects of pulse width (t_on_) on the machining performance, the effective voltage time (t_effv_) was calculated as the summation of t_on_ over t_dwell_, in which case t_effv_ could be set to a constant with t_on_ varying.

The cross-sectional profiles and geometric parameters of the machined dimples were obtained from the laser scanning confocal microscope (LSCM), KEYENCE VK-X2000. The LSCM employed to measure surface roughness (Sa) and waviness (Wa) was the VKX2000 supplied by KEYENCE. The dimple morphology was observed by the scanning electron microscope (SEM), Quanta FEG 450. The elemental analysis was conducted by energy-dispersive spectrometry (EDS), Supra 55 Sapphire.

## 3. Results and Discussions

In this section, the impact of the voltage, effective voltage time and duty cycle on the machining performance is evaluated by comparing the morphology, the diameter and the depth of dimples. The scale bar of the pictures below is 50 μm.

### 3.1. The Effect of Voltage

#### 3.1.1. Morphologies

**Figure 2:**
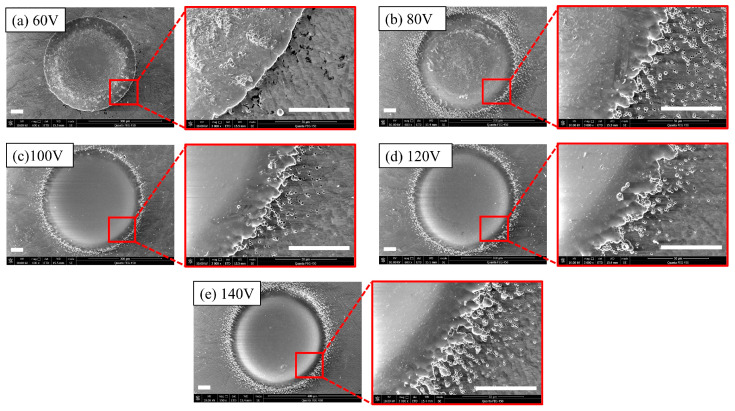
SEM morphologies at different voltages (the scale bar is 50 μm): (**a**) 60 V; (**b**) 80 V; (**c**) 100 V; (**d**) 120 V; (**e**) 140 V.

In order to assess the impact of voltage on the machining effectiveness while keeping other parameters constant, a 200 μm diameter nozzle was used The voltage was sequentially adjusted from 60 V to 140 V, as shown in Table 1. The SEM pictures corresponding to various processing parameters are shown in Figure 2. With the increase in voltage, the area of edge scatter corrosion expanded. With an applied voltage of 140 V, the stray corrosion area approached approximately 40 μm. Under the voltage of 60 V, the edge exhibited minimal stray corrosion and virtually no occurrence of pitting, but surface irregularities were present. As the voltage increased to 100 V, the machining surface quality significantly improved. Under an applied voltage of 60 V, the surface roughness was 1.064 μm. When the voltage increased to 80 V or 100 V, the surface roughness approached 0.55 μm. Raising the voltage further increased the surface roughness. When the voltage was set to 140 V, the surface roughness was 0.83 μm, which was still lower than the surface roughness at 60 V. The most likely reason for this trend is the change of current density. When the applied voltage was 60 V, the current density was only 21.3 A/cm^2^. With an increase in the voltage to 100 V, the current density reached 33.7 A/cm^2^, allowing for the removal of materials, causing surface irregularities and achieving a smooth surface finish. As for the pitting appearing at the edges of the machined dimples, the most likely cause is the influence of machining depth. With the increase in applied voltage, the depth of the dimples also increased. When the electrolyte is injected into the dimples, the greater depth of the dimples makes it challenging for the liquid to be expelled, which can lead to splashing and the formation of pitting.

#### 3.1.2. Geometric Characterization

**Figure 3:**
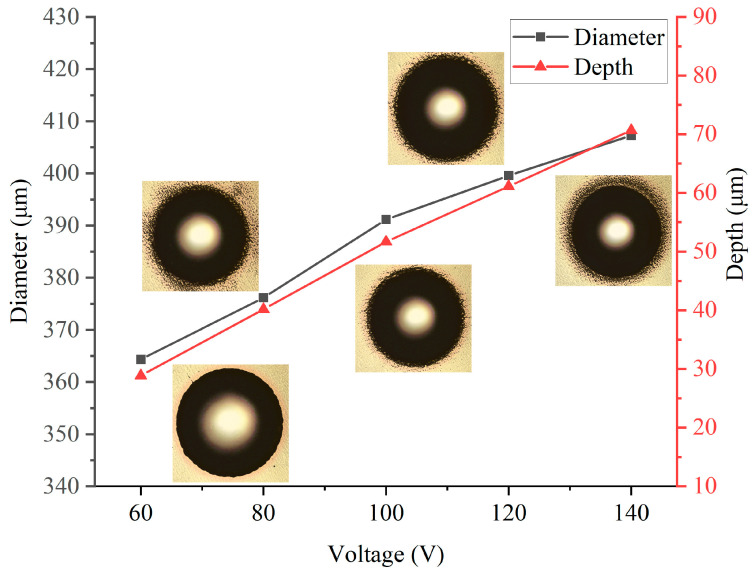
Diameters and depths at different voltages.

The diameter and depth of the dimples are shown in Figure 3. It is evident that as the applied voltage increased, both the depth and diameter of the dimples also increased. This indicates that the material removal rate increased with the rising applied voltage. The shaded areas in the pictures are the measurement errors caused by the steepness of the dimples’ wall. As the applied voltage increased, the dimples also became larger, indicating that the taper angle of the machined dimples was also increasing. At the applied voltage of 60 V, the diameter of the dimple approached approximately 365 μm, which was about 1.8 times the diameter of the nozzle (200 μm). This phenomenon can be explained by the fact that as the applied voltage increased, the current density at the edge of the liquid column also increased. Consequently, anodic dissolution occurred at the edge location. When increasing the applied voltage from 60 V to 140 V, the dimple diameter expanded from 360 μm to 405 μm, resulting in a 45 μm increase. Therefore, adjusting the applied voltage unilaterally to achieve deeper dimples is not advisable, as it may lead to more severe stray corrosion or undesirable shapes. It is essential to coordinate with other machining parameters for better results.

### 3.2. The Effect of Duty Cycles

#### 3.2.1. Morphologies

**Figure 4:**
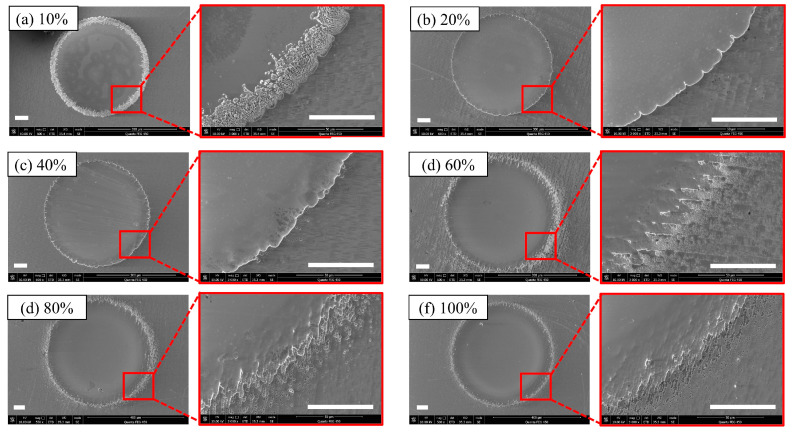
Diameters and depths at different duty cycles (the scale bar is 50 μm): (**a**) 10% (t_on_ = 20 μs); (**b**) 20% (t_on_ = 40 μs); (**c**) 40% (t_on_ = 80 μs); (**d**) 60% (t_on_ = 120 μs); (**e**) 80% (t_on_ = 160 μs); (**f**) 100% (t_on_ = 200 μs).

Under the condition (Table 2) that t_effv_ is consistent, the dimples obtained by changing the duty cycle are shown in Figure 4. The roundness of the dimples produced under various machining conditions appeared to be consistent with no significant differences. In Figure 4a, it can be observed that loose material features appear at the dimple edges when t_on_ = 20 μs. This phenomenon can be explained by the fact that under very low-pulse-width machining conditions, the voltage between the cathode and anode could not be sustained for a sufficient duration to enable anodic dissolution. Additionally, the low current density at dimple edges during the machining process was insufficient for complete electrolysis within a short timeframe. When t_on_ = 40 μs and t_on_ = 80 μs, this situation showed significant improvement with minimal stray corrosion and sporadic pitting. However, when t_on_ increased to 120 μs, noticeable stray corrosion began to appear, as shown in Figure 4d, and it became most pronounced at t_on_ = 160 μs, reaching a maximum of nearly 30 μm. When t_on_ = 200 μs, there was some improvement in the aspect of stray corrosion. The most likely explanation for this phenomenon is the extended electrolytic processing region, causing the solution to affect the stray corrosion area closer to the center, while the stray corrosion area farther from the center remained unaffected. Additionally, it can be observed that under the consistent t_effv_, varying the pulse width time did not lead to significant differences in terms of the dimple surface. The dimples under varying duty cycles displayed a flat appearance with regular profiles.

#### 3.2.2. Geometric Characterization

**Figure 5:**
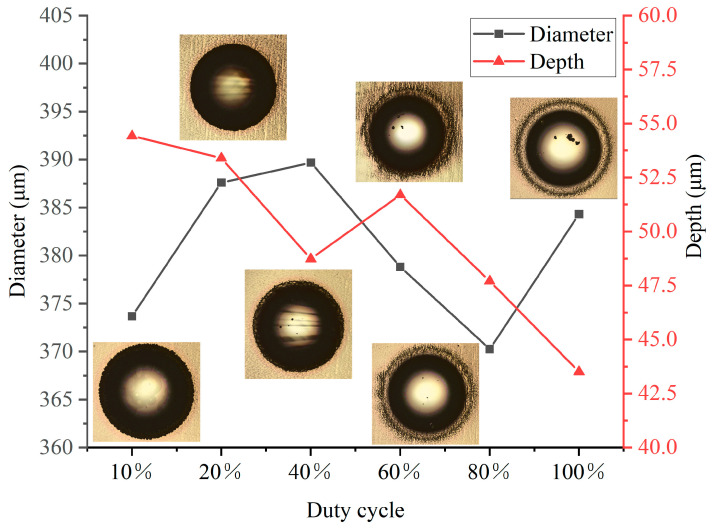
Diameters and depths at different duty cycles.

It can be observed in Figure 5 that, under the condition of the consistent t_effv_, the depth and diameter of the dimple were not directly proportional to the pulse width. Under the condition of t_on_ = 20 μs, the diameter of the dimple was approximately 375 μm, and it had the greatest depth among all the experimental groups. This phenomenon can be explained by the fact that with the minimum duty cycle, the electrode remains energized for the longest duration, allowing the center of the jet column to maintain a voltage that enabled anodic dissolution for an extended period. As a result, the change in current density was relatively slow, allowing for the attainment of deeper dimples. With an increase in the duty cycle, there was a general decrease in the dimple depth. When t_on_ = 200μs, the depth of the dimple was minimal. One possible reason is that the continuous electrolysis resulted in the untimely removal of dissolved products, hindering further reactions. Therefore, to achieve a greater depth while maintaining the desired external contour, it is necessary to modify both voltage and duty cycle. Subsequent research will focus on comparing these two parameters. Moreover, it can be observed that the maximum surface roughness at the center of the dimples occurred at t_on_ = 20 μs, reaching 1.07 μm. When t_on_ = 200 μs, the surface roughness was 0.55 μm. The surface quality improved with the increase of t_on_. This trend highlights the influence of t_on_ on the surface quality. Therefore, it is possible to achieve a lower surface roughness by increasing the duty cycle.

### 3.3. The Effect of Effective Voltage Time

#### 3.3.1. Morphologies

**Figure 6:**
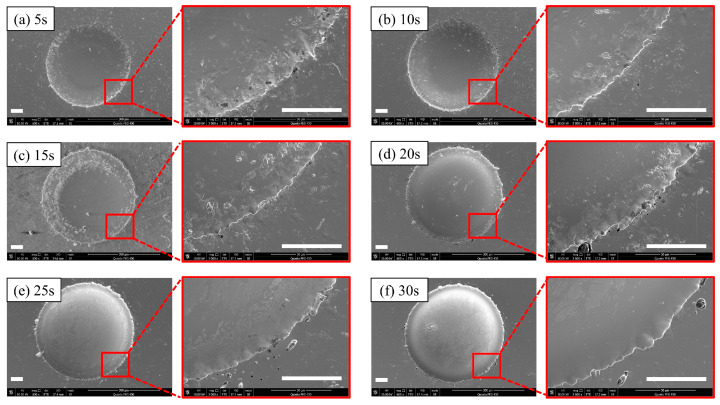
Diameters and depths at different effective voltage times (the scale bar is 50 μm): (**a**) t_effv_ = 5 s; (**b**) t_effv_ = 10 s; (**c**) t_effv_ = 15 s; (**d**) t_effv_ = 20 s; (**e**) t_effv_ = 25 s; (**f**) t_effv_ = 30 s.

By analyzing the impact of the duty cycle on the machining results (Table 3), it is evident that there was less stray corrosion with duty cycles of 20% and 40%. Therefore, the duty cycle of 40% was used in the subsequent experiments. When maintaining other parameters at a consistent level, the effective voltage time increased with the space of 5 s, starting from the duration of 5 s. As shown in Figure 6, when t_effv_ = 5 s, the stray corrosion was not prominent, and relatively regular shapes could be maintained. As the t_effv_ increased, the stray corrosion became more apparent. When the t_effv_ extended to 25 s, the stray corrosion started to decrease, but it re-emerged as a more severe issue when the t_effv_ increased to 30 s. The surface quality at the center showed no evident difference. This phenomenon can be attributed to the higher current density and strong electrolyte flushing, resulting in a more uniform dissolution at the center. Additionally, at t_effv_ = 15 s, surface irregularities appeared in regions extending from the center to the edges. With the further increase in t_effv_, the aforementioned phenomenon showed improvement. This can be explained by the fact that farther from the center, the current density was lower, and it took sufficiently long time to achieve partial anodic dissolution. Therefore, the use of an inappropriate t_effv_ could lead to irregularities in the transition area.

#### 3.3.2. Geometric Characterization

**Figure 7:**
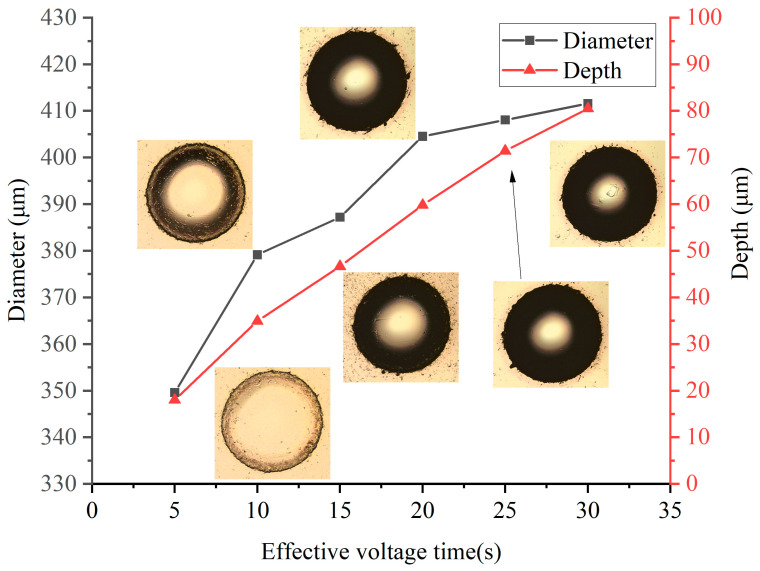
Diameters and depths at different effective voltage times.

In Figure 7, a noticeable increase in both the dimple diameter and depth can be observed as the t_effv_ increased. When t_effv_ = 5 s, the diameter of dimple was 350 μm, which was 1.75 times the nozzle diameter, with the central depth ranging from 15 μm to 20 μm. With an increase in effective voltage time to t_effv_ = 30 s, the diameter of dimple approached 410 μm, which was 2.05 times the nozzle diameter, and the central dimple depth increased from 80 μm to 83 μm. Furthermore, at t_effv_ = 5 s, the dimples displayed a relatively bright surface when subjected to LSCM scanning, with clearly visible transition areas at the edges. With an increase of t_effv_, black-shadowed regions emerged at the dimple edges, primarily due to image capture inaccuracies, signifying an intensified dimple conicity. Surface roughness measurements at the dimple center, conducted with LSCM, showed that when the t_effv_ ranged from 5 s to 25 s, the center surface roughness remained stable, ranging from 0.8 μm to 0.85 μm. However, when t_effv_ = 30 s, a significant surface irregularity became apparent at the dimple center, with the surface roughness of 1.08 μm. This phenomenon can be explained by the fact that, after a certain duration of electrolytic processing, the depth of the dimple increased considerably. In regions with reduced current density, achieving uniform further reactions becomes challenging. Additionally, the presence of persistent bubbles in deeper regions makes it difficult to maintain a smooth surface, resulting in the observed irregularities.

**Figure 8:**
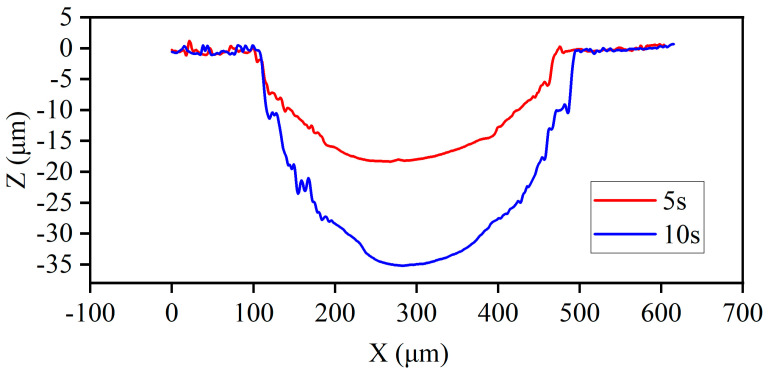
Cross-section profiles under different effective voltage times.

Figure 8 displays the cross-sectional profiles of two sets of machining parameters. It can be observed that as the t_effv_ increased from 5 s to 10 s, the depth of the dimples increased from 15 μm to 35 μm, and both maintained a consistent and regular profile. However, when t_effv_ = 10 s, the surface became rough. Additionally, it can be observed that with the increase in t_effv_, the greatest change in depth occurred at the center of the dimples. This indicates that during the machining process, the current density at the center of the jet column was the highest and gradually decreased toward the edges. Another possibility is that the intense jetting at the center accelerated the dissolution of the material at the center.

#### 3.3.3. Element Content Analysis Results

**Figure 9:**
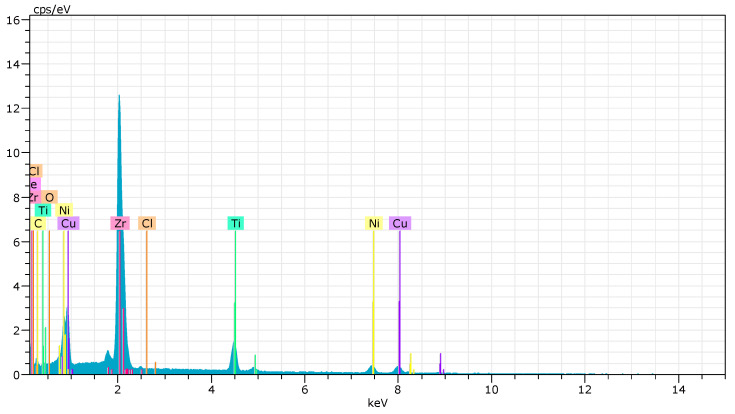
Element content at t_effv_ = 10 s.

Quantitative characterization of the surface composition was performed as shown in Table 4 and Figure 9. The machining parameters included a pulse cycle of 200 μs, a duty cycle of 50%, an applied voltage of 100 V, an initial gap of 200 μm and a flow rate of 52 mL/min. Results were obtained by varying t_effv_ for numerical comparisons. According to the results, the surface elemental ratios after jet-ECM were very similar to the original surface, with all samples containing less O and C than the original surface. This suggests that using a NaCl-EG electrolyte is effective in removing the amorphous layer, achieving uniform electrochemical dissolution and maintaining an unchanged surface element composition.

## 4. Conclusions

This research employed jet electrochemical machining to perform material removal and create dimples on Zr- BMGs. In order to reduce the formation of dense and adherent oxide layers caused by the use of water-based salt electrolytes, alcohol-based electrolytes were employed. The feasibility of this approach was explored through the characterization of the machined dimples. The research primarily compared and analyzed the effects of voltage, effective voltage time and duty cycle. The main conclusions are summarized as follows:

(1) The experimental results showed that Zr-based BMGs can be processed effectively by high-flow-rate electrochemical processing. Because the current density of the machining center was larger than that of the edge, it was evident from the cross-section profiles that the center of the dimple had the greatest depth. Compared with the original surface, the changes of each component after processing were not obvious. In addition, using the NaCl-EG electrolyte and spraying the electrolyte at a high flow rate could reduce the generation of a passivation film.
(2) The experimental results demonstrated that increasing the applied voltage and extending the effective voltage time both contributed to enlarging the diameter and depth of the dimples to some extent. However, there was also a notable increase in the stray corrosion. However, the increase in the duty cycle did not exhibit such a consistent pattern. It is crucial to select the appropriate machining parameters to achieve optimal results. Micro dimples with less stray corrosion can be fabricated using jet-ECM with a cycle of 200 μs and a duty cycle of 20% and 40%. Increasing the voltage properly can obtain deeper and wider dimples and make the surface flatter, but it is necessary to pay attention to the stray corrosion caused by this.
(3) Under appropriate parameters, it is possible to achieve a relatively smooth surface and significantly reduce stray corrosion. Subsequent research will focus on the impact of the initial gap and nozzle diameter on machining outcomes. In addition, different nozzle shapes will be designed to obtain more diverse processing effects.

## Figures and Tables

**Table 1:** Experimental parameters of the voltage group.

Voltage (V)	t_cyc_ (μs)	Duty Ratio (%)	t_effv_ (s)	Gap (μm)	Flow Rate (mL/min)
60, 80, 100, 120,140	200	50	15	200	52

**Table 2:** Experimental parameters of the duty cycle group.

Voltage (V)	t_cyc_ (μs)	Duty Cycle (%)	t_effv_ (s)	Gap (μm)	Flow Rate (mL/min)
100	200	10, 20, 40 60, 80, 100	15	200	52

**Table 3:** Experimental parameters of the effective voltage time group.

Voltage (V)	t_cyc_ (μs)	Duty Cycle (%)	t_effv_ (s)	Gap (μm)	Flow Rate (mL/min)
100	200	40	5, 10, 15, 20, 25, 30	200	52

**Table 4:** Comparison of the main element content.

Jet-ECM Conditions	Be (%)	C (%)	O (%)	Ti (%)	Ni (%)	Cu (%)	Zr (%)
Initial surface	2.02	18.32	5.53	13.45	7.76	11.32	41.45
t_effv_ = 5 s	1.04	15.08	1.6	16.29	9.83	11.09	45.36
t_effv_ = 10 s	4.12	16.43	0.4	14.58	8.98	11.34	44.29
t_effv_ = 15 s	4.41	14.67	1.96	15.28	948	11.34	42.89

## Data Availability

The data presented in this study are openly available.
